# Supplementary material for: Effect of Lactobacillus acidophilus, Oenococcus oeni, and Lactobacillus brevis on Composition of Bog Bilberry Juice
Source: Foods. 2019 Sep 21;8(10):430. doi: 10.3390/foods8100430 (PMC6835820; doi:10.3390/foods8100430)
Supplement: Supplementary file 1 [file foods-08-00430-s001.pdf]

Supplementary

Table S1. Values of bacterial growth ratio in bog bilberry juice after inoculating with three lactic acid bacteria strains in 14 days.

Strain	Time (Day)					
	0	1	2	3	7	14
L. brevis	1.000±0.004 Ad	0.799±0.031 Ab	0.648±0.004 Aa	0.799±0.012 Ab	0.860±0.025 Bc	0.903±0.004 Bc
L. acidophilus	1.000±0.003 Ad	0.998±0.013 Bd	0.951±0.013 Cc	0.945±0.010 Cc	0.821±0.006 Ab	0.752±0.015 Aa
O. oeni	1.000±0.003 Ac	0.869±0.018 Ab	0.747±0.005 Ba	0.860±0.009 Bb	1.034±0.004 Cc	1.022±0.022 Cc

Data are mean ± standard deviation of triplicate tests; Different letters in lower case indicate significant differences with time, whereas different letters in upper case represent significant difference in juice inoculated with different strains at the same sampling interval at $P<0.05$.

Table S2. Content of reducing sugar, organic acid and pH in bog bilberry juice inoculated with three lactic acid bacteria strains in 14 days.

Compound	Strain	Time (Day)					
		0	1	2	3	7	14
Reducing sugars (g/L)							
Glucose	*L. brevis*	11.49±0.12 Ac	11.51±0.77 Ac	11.05±0.07 Bc	9.45±0.15 Ab	8.54±0.49 Ab	4.82±0.02 Aa
	L. acidophilus	11.49±0.12 Ac	10.7±0.62 Ac	10.76±0.64 ABc	10.65±0.52 Bc	8.09±0.29 Ab	4.14±0.8 Aa
	O. oeni	11.49±0.12 Ad	10.16±0.16 Ac	9.81±0.32 Ac	9.52±0.36 Ac	7.87±0.19 Ab	4.02±0.27 Aa
Fructose	*L. brevis*	12.28±0.10 Ac	11.13±0.5 Abc	11.58±0.52 Abc	11.05±0.73 Abc	10.55±0.66 Ab	9.1±0.2 Aa
	L. acidophilus	12.28±0.10 Ac	11.56±0.5 Abc	11.25±0.23 Abc	11.49±0.42 Abc	10.56±0.19 Ab	7.97±0.8 Aa
	O. oeni	12.28±0.10 Ad	10.92±0.16 Ac	10.66±0.29Ac	10.86±0.17 Ac	9.84±0.12 Ab	8.46±0.32 Aa
Total	*L. brevis*	23.77±0.22 Ac	22.64±0.68 Ac	22.63±0.54 Bc	20.5±0.75 Ab	19.09±1.14 Ab	13.92±0.22 Aa
	L. acidophilus	23.77±0.22 Ac	22.25±1.11 Ac	22.01±0.56 Bc	22.14±0.94 Ac	18.65±0.48 Ab	12.11±1.6 Aa
	O. oeni	23.77±0.22 Ad	21.08±0.32 Ac	20.47±0.61 Ac	20.38±0.47 Ac	17.72±0.31 Ab	12.48±0.48 Aa
Organic acids (g/L)							
Quinic acid	*L. brevis*	6.50±0.34 Aab	5.86±0.30 Aa	6.93±0.04 Aab	7.29±0.63 Aab	7.48±0.22 Ab	6.21±0.31 Aab
	L. acidophilus	6.50±0.34 Aa	7.70±1.06 Aa	7.63±0.17 Aa	7.44±0.11 Aa	6.90±0.12 Aa	6.35±0.39 Aa
	O. oeni	6.50±0.34 Aa	5.96±0.69 Aa	7.03±0.31 Aa	6.61±0.18 Aa	7.04±0.24 Aa	5.90±0.30 Aa
Malic acid	*L. brevis*	3.22±0.10 Aa	3.54±0.35 Aa	3.15±0.07 Aa	3.13±0.02 Ba	2.95±0.12 Aa	3.03±0.07 Aa
	L. acidophilus	3.22±0.10 Ac	2.64±0.07 Aa	2.96±0.05 Abc	2.97±0.05 Abc	2.83±0.07 Aab	3.19±0.11 Ac
	O. oeni	3.22±0.10 Aa	3.22±0.07 Ba	2.99±0.12 Aa	2.99±0.06 Aa	2.90±0.03 Aa	3.11±0.32 Aa
Citric acid	*L. brevis*	2.94±0.11 Aa	2.74±0.13 Aa	2.75±0.04 Aa	2.93±0.02 Aa	2.95±0.15 Aa	2.91±0.19 Aa
	L. acidophilus	2.94±0.11 Aa	2.69±0.30 Aa	3.14±0.13 Ba	3.00±0.03 Aa	2.92±0.02 Aa	3.28±0.24 Aa
	O. oeni	2.94±0.11 Ab	2.17±0.21 Aa	2.73±0.12 Ab	2.88±0.10 Ab	2.64±0.02 Ab	2.67±0.08 Ab
Lactic acid	*L. brevis*	nd	nd	nd	nd	nd	nd
	L. acidophilus	nd	0.31±0.04 Aa	0.40±0.07 Aa	0.50±0.03 Aab	0.77±0.14 Ac	0.75±0.08 Abc
	O. oeni	nd	nd	nd	nd	nd	nd

Oxalic acid	*L. brevis*	0.05±0.01 Aa	0.06±0.00 Aa	0.08±0.01 Aab	0.09±0.02 Aab	0.12±0.03 Ab	0.05±0.01 Aa
	L. acidophilus	0.05±0.01 Aa	tr	0.06±0.00 Aa	0.11±0.01 Ab	0.08±0.01 Aab	0.06±0.03 Aa
	O. oeni	0.05±0.01 Aa	0.04±0.00 Aa	0.10±0.01 Bb	0.10±0.01 Ab	0.11±0.02 Ab	0.05±0.02 Aa
Shikimic acid	*L. brevis*	0.04±0.00 Aa	0.04±0.01 Aa	0.03±0.00 Aa	0.03±0.00 Aa	0.04±0.00 Aa	0.03±0.00 Aa
	L. acidophilus	0.04±0.00 Ab	0.02±0.00 Aa	0.03±0.00 Aab	0.03±0.00 Aab	0.03±0.00 Aab	0.03±0.00 Aab
	O. oeni	0.04±0.00 Aa	0.03±0.00 Aa	0.03±0.00 Aa	0.04±0.00 Aa	0.03±0.00 Aa	0.03±0.00 Aa
Total	*L. brevis*	12.74±0.98 Aa	12.24±0.73 Aa	12.93±0.12 Aa	13.47±0.63 ABa	13.53±0.44 Aa	12.24±0.47 ABa
	L. acidophilus	12.74±0.98 Aa	13.37±0.79 Aa	14.22±0.30 Ba	14.06±0.18 Ba	13.54±0.30 Aa	13.66±0.35 Ba
	O. oeni	12.74±0.98 Aa	11.43±0.83 Aa	12.89±0.54 Aa	12.62±0.20 Aa	12.73±0.27 Aa	11.75±0.62 Aa
pH							
pH	*L. brevis*	2.66±0.00 Ab	2.67±0.01 Ab	2.67±0.01 Ab	2.66±0.01 Ab	2.65±0.02 Ab	2.62±0.00 Aa
	L. acidophilus	2.66±0.00 Abc	2.68±0.01 Ac	2.68±0.00 Abc	2.66±0.01 Abc	2.65±0.00 Ab	2.62±0.01 Aa
	O. oeni	2.66±0.00 Ab	2.70±0.00 Acd	2.70±0.01 Bd	2.68±0.01 Abcd	2.67±0.02 Abc	2.62±0.01 Aa

Data are mean ± standard deviation of triplicate tests "nd" represents "not detected"; Different letters in lower case indicate significant differences with time, whereas different letters in upper case represent significant difference in juice inoculated with different strains at the same sampling interval at $P<0.05$.

Table S3. Content of amino acids, ammonium ion, and biogenic amines in bog bilberry juice inoculated with three lactic acid bacteria strains in 14 days.

Compound	Strain	Time (Day)					
(mg/L)		0	1	2	3	7	14
Amino acids							
Valine	*L. brevis*	14.26±0.31 Ac	13.95±0.29 Ac	18.48±0.34 Bd	14.88±1.36 Ac	4.58±0.63 Ab	0.10±0.22 Aa
	L. acidophilus	14.26±0.31 Ac	16.78±1.21 Ad	20.07±0.52 Ce	19.79±0.48 Be	5.82±0.72 Ab	0.77±0.00 Ba
	O. oeni	14.26±0.31 Ab	14.38±0.65 Ab	14.68±0.28 Ab	18.25±1.36 ABc	4.39±0.60 Aa	tr
Asparaginate	*L. brevis*	10.23±0.93 Ab	7.35±0.42 Ab	1.91±2.29 Aa	0.92±0.11 Aa	1.44±0.25 Aa	0.02±0.04 Aa
	L. acidophilus	10.23±0.93 Ac	7.75±0.27 Abc	10.20±0.77 Bc	6.03±0.71 Bb	1.90±2.28 Aa	0.25±0.10 Aa
	O. oeni	10.23±0.93 Ab	8.87±0.92 Ab	8.78±0.90 Bb	0.92±0.00 Aa	1.23±0.06 Aa	0.61±0.07 Ba
Glutamine	*L. brevis*	6.28±0.08 Ab	5.80±0.32 Aab	8.96±4.50 Aab	3.16±3.05 Aab	0.78±0.34 Aab	0.59±0.07 Aa
	L. acidophilus	6.28±0.08 Aab	11.69±0.67 Bcd	13.04±0.90 Ad	9.00±1.28 Abc	4.73±0.33 Ba	3.82±1.34 Ba
	O. oeni	6.28±0.08 Ac	5.93±0.26 Ac	5.80±0.21 Ac	4.30±0.00 Ab	0.40±0.10 Aa	0.33±0.03 Aa
Arginine	*L. brevis*	6.61±0.19 Ad	2.54±0.25 Bc	1.73±0.98 Ac	0.64±0.03 Ab	0.48±0.14 Aa	0.16±0.01 Aa
	L. acidophilus	6.61±0.19 Ad	6.10±0.16 Cc	5.35±0.43 Bc	3.94±0.90 Bb	0.29±0.00 Aa	0.38±0.03 Ba
	O. oeni	6.61±0.19 Ac	1.37±0.04 Ab	1.02±0.08 Ab	0.74±0.34 Aa	0.26±0.05 Aa	0.21±0.01 Aa
Serine	*L. brevis*	3.80±0.71 Abc	4.26±0.14 Bc	4.61±0.20 Cc	2.90±0.53 Bb	0.54±0.03 Ba	0.43±0.03 ABa
	L. acidophilus	3.80±0.71 Acd	4.52±0.17 Bd	3.39±0.03 Bc	1.50±0.08 Ab	0.23±0.07 Aa	1.01±0.33 Bb
	O. oeni	3.80±0.71 Ad	2.80±0.06 Ac	2.61±0.19 Ac	1.49±0.00 Ab	tr	0.18±0.05 Aa
Phenylalanine	*L. brevis*	2.21±0.06 Ab	0.74±0.81 Aa	tr	tr	tr	tr
	L. acidophilus	2.21±0.06 Ab	1.45±0.96 Aab	0.30±0.09 Aab	tr	0.01±0.08 Aa	2.00±0.91 Ab
	O. oeni	2.21±0.06 Aab	4.80±2.34 Ab	1.98±0.01 Bab	0.65±0.30 Aa	1.35±0.03 Bab	tr
Tryptophan	*L. brevis*	1.23±0.43 Ab	0.50±0.35 Aa	tr	1.06±0.44 Bb	tr	tr
	L. acidophilus	1.23±0.43 Ab	tr	tr	tr	tr	0.05±0.07 Aa
	O. oeni	1.23±0.43 Abc	2.23±0.34 Bd	1.84±0.36 Acd	0.37±0.27 ABab	0.40±0.09 Aab	tr
Glycine	*L. brevis*	3.28±0.03 Ab	2.97±0.06 ABb	3.02±0.06 ABb	2.79±0.14 Ab	0.74±0.31 Aa	0.50±0.00 Ba
	L. acidophilus	3.28±0.03 Ac	3.19±0.04 Bc	3.21±0.07 Bc	2.51±0.37 Abc	1.65±0.69 Ab	0.37±0.01 Aa
	O. oeni	3.28±0.03 Ad	2.82±0.10 Ac	2.75±0.12 Ac	1.99±0.05 Ab	0.42±0.01 Aa	0.32±0.00 Aa

Histidine	*L. brevis*	1.75±0.03 Abc	1.49±0.09 Ab	2.38±0.54 Bc	1.38±0.11 Bb	0.41±0.09 Aa	0.13±0.03 Aa
	L. acidophilus	1.75±0.03 Ad	1.43±0.10 Ad	0.87±0.04 Ac	0.70±0.10 Abc	0.40±0.19 Aab	0.31±0.07 Ba
	O. oeni	1.75±0.03 Ae	1.48±0.03 Ad	1.04±0.01 Ac	0.71±0.03 Ab	0.14±0.01 Aa	0.16±0.00 Aa
Lysine	*L. brevis*	4.00±0.00 Ac	3.09±0.41 Abc	3.54±0.21 Ac	1.99±0.87 Ab	0.13±0.13 Aa	0.02±0.01 Aa
	L. acidophilus	4.00±0.00 Ab	3.95±0.10 Ab	3.75±0.31 Ab	2.38±0.81 Aab	1.21±1.25 Aa	2.06±1.03 Aab
	O. oeni	4.00±0.00 Ac	2.70±1.17 Abc	3.38±0.35 Abc	1.61±0.98 Aab	0.16±0.01 Aa	0.07±0.03 Aa
Methionine	*L. brevis*	3.93±1.75 Ab	3.67±0.17 Ab	3.33±0.49 Ab	1.68±0.03 Aa	0.79±0.36 Aa	1.41±0.08 Aa
	L. acidophilus	3.93±1.75 Ab	3.44±0.34 Ab	4.13±0.49 Ab	2.78±0.06 Ba	2.41±0.10 Ba	1.12±0.70 Aa
	O. oeni	3.93±1.75 Ac	3.20±0.04 Abc	3.01±0.22 Abc	2.98±0.27 Bbc	2.22±0.30 Bab	1.48±0.13 Aa
Ornithine	*L. brevis*	0.38±0.00 Aa	2.19±0.21 Bb	2.56±0.20 Bb	1.95±0.22 Bb	0.45±0.22 Aa	0.21±0.00 ABa
	L. acidophilus	0.38±0.00 Aab	0.35±0.01 Aab	0.44±0.05 Ab	0.41±0.02 Ab	0.20±0.10 Aa	0.35±0.07 Bab
	O. oeni	0.38±0.00 Aa	3.79±0.43 Cb	4.10±0.12 Cb	3.99±0.10 Cb	0.25±0.00 Aa	0.16±0.01 Aa
Leucine	*L. brevis*	2.14±0.18 Aab	3.62±1.06 Abc	4.63±0.29 Ac	3.46±0.70 Abc	1.60±0.53 Aab	0.16±0.00 Aa
	L. acidophilus	2.14±0.18 Aab	2.97±0.86 Ab	3.79±0.30 Ab	3.31±0.72 Ab	2.27±0.91 Aab	0.38±0.08 Ba
	O. oeni	2.14±0.18 Ab	2.09±0.01 Ab	1.74±0.19 Bb	1.66±0.83 Ab	0.41±0.03 Aa	0.16±0.00 Aa
γ-Aminobutyric acid	*L. brevis*	3.09±0.05 Ad	2.92±0.04 Ac	3.44±0.03 ABd	2.70±0.41 Ac	0.63±0.04 Ab	0.27±0.06 Aa
	L. acidophilus	3.09±0.05 Ac	3.16±0.10 Ac	3.53±0.08 Bd	3.07±0.15 Ac	0.61±0.03 Aa	1.19±0.14 Bb
	O. oeni	3.09±0.05 Ac	3.06±0.12 Ac	3.23±0.11 Ac	3.05±0.19 Ac	0.62±0.05 Ab	0.39±0.02 Aa
Isoleucine	*L. brevis*	0.28±0.01 Aa	1.10±0.04 Aab	1.09±0.13 Bab	0.18±0.20 Aa	1.16±0.77 Aab	2.48±0.64 Bb
	L. acidophilus	0.28±0.01 Aa	1.33±0.14 Aab	1.39±0.17 Bb	0.86±0.31 Aab	0.52±0.45 Aab	0.61±0.40 Aab
	O. oeni	0.28±0.01 Aab	0.77±0.46 Ab	0.26±0.02 Aab	0.25±0.26 Aab	0.12±0.01 Aa	tr
Tyrosine	*L. brevis*	2.79±0.03 Abc	2.28±0.25 Bb	3.45±0.38 Bc	2.58±0.43 Abc	2.43±0.23 Ab	1.30±0.29 Ba
	L. acidophilus	2.79±0.03 Ab	0.57±0.53 Aa	0.86±0.82 Aa	0.42±0.04 Aa	0.34±0.30 Aa	0.56±0.17 Aa
	O. oeni	2.79±0.03 Ac	1.75±0.15 ABa	1.70±0.04 ABa	1.39±0.12 Ba	1.55±0.26 Aa	2.65±0.23 Cb
Cysteine	*L. brevis*	2.20±0.15 Ab	2.31±0.07 Bb	2.39±0.32 Ab	2.20±0.25 Ab	0.71±0.52 Aa	1.86±0.05 Ab
	L. acidophilus	2.20±0.15 Aab	2.35±0.26 Bab	2.54±0.17 Ab	2.02±0.03 Aa	2.01±0.02 Ba	2.11±0.01 Aab
	O. oeni	2.20±0.15 Ab	0.80±0.61 Aa	2.08±0.00 Ab	2.25±0.09 Ab	2.16±0.02 Bb	1.88±0.17 Ab
Proline	*L. brevis*	1.41±0.24 Ab	0.50±0.00 Aa	0.79±0.13 Aa	1.32±0.08 Ab	1.44±1.44 Cb	tr

	L. acidophilus	1.41±0.24 Aa	1.14±0.16 Ba	1.92±0.06 Cb	1.30±0.02 Aa	1.21±0.03 Ba	tr
	O. oeni	1.41±0.24 Ac	1.25±0.02 Bbc	1.21±0.03 Bbc	1.23±0.18 Abc	1.03±0.05 Aab	0.81±0.05 Aa
β-Alanine	*L. brevis*	0.85±0.01 Ac	0.89±0.06 Ac	1.00±0.02 Bd	0.44±0.01 Ab	0.06±0.01 Aa	0.12±0.03 Ba
	L. acidophilus	0.85±0.01 Ac	0.88±0.02 Ac	0.83±0.05 Ac	0.47±0.21 Ab	0.10±0.01 Aa	0.08±0.01 ABa
	O. oeni	0.85±0.01 Ab	0.83±0.04 Ab	0.71±0.05 Ab	0.06±0.06 Aa	0.11±0.04 Aa	0.04±0.01 Aa
Threonine	*L. brevis*	1.33±0.02 Ac	1.24±0.00 Ac	1.33±0.03 Abc	1.17±0.07 Ab	0.87±0.03 Aa	0.80±0.00 Aa
	L. acidophilus	1.33±0.02 Ac	1.30±0.00 Bc	1.33±0.02 Ac	1.22±0.03 Ab	0.88±0.00 Aa	0.89±0.01 Ba
	O. oeni	1.33±0.02 Ac	1.27±0.02 ABbc	1.27±0.01 Abc	1.21±0.05 Ab	0.86±0.01 Aa	0.89±0.00 Ba
Aspartic acid	*L. brevis*	0.52±0.00 Abc	0.58±0.02 Bcd	0.64±0.01 Bd	0.46±0.03 Ab	0.02±0.03 Aa	0.06±0.01 Aa
	L. acidophilus	0.52±0.00 Ab	0.72±0.02 Cc	0.76±0.03 Cc	0.66±0.03 Bc	0.48±0.09 Cb	0.04±0.02 Aa
	O. oeni	0.52±0.00 Ac	0.40±0.03 Abc	0.39±0.03 Ab	0.52±0.07 ABc	0.22±0.03 Ba	tr
Glutamic acid	*L. brevis*	0.65±0.03 Ac	0.50±0.05 Ab	0.65±0.05 Ac	0.67±0.05 Ac	0.51±0.02 ABb	0.15±0.02 Aa
	L. acidophilus	0.65±0.03 Ac	0.61±0.05 ABc	0.71±0.04 Ac	0.67±0.04 Ac	0.47±0.04 Ab	0.20±0.03 Aa
	O. oeni	0.65±0.03 Aabc	0.66±0.02 Bbc	0.69±0.02 Ac	0.65±0.00 Aabc	0.61±0.03 Bab	0.58±0.03 Ba
α-Alanine	*L. brevis*	0.06±0.01 Ab	0.07±0.00B Ac	0.05±0.00 Ab	0.05±0.00 Ab	0.06±0.00 Bb	0.03±0.01 Aa
	L. acidophilus	0.06±0.01 Abc	0.07±0.01 Bc	0.04±0.01 Aab	0.04±0.00 Aab	0.03±0.00 Aa	0.03±0.00 Aa
	O. oeni	0.06±0.01 Aa	0.03±0.01 Aa	0.06±0.01 Aa	0.04±0.02 Aa	0.04±0.01 AB	0.04±0.01 Aa
Total	*L. brevis*	73.28±3.32 Ad	64.56±4.64 Acd	69.98±9.86 Ad	48.58±6.16 Ac	19.83±5.53 Ab	10.80±1.45 Aa
	L. acidophilus	73.28±3.32 Abc	73.47±5.44 Abc	82.45±4.76 Bc	63.08±5.55 Bb	27.77±5.81 Ba	18.58±5.19 Aa
	O. oeni	73.28±3.32 Ad	66.47±5.93 Ad	64.33±2.86 Ad	50.31±5.26 Ac	18.95±1.50 Ab	10.96±0.73 Aa
Ammonium amine							
NH_4^+	*L. brevis*	2.08±1.76 Ab	3.04±0.07 Ac	3.14±0.02 Ac	1.18±0.59 Aa	tr	tr
	L. acidophilus	2.08±1.76 Abc	3.23±0.73 Ac	3.03±0.85 Ac	1.43±0.46 Abc	0.30±1.03 Aab	tr
	O. oeni	2.08±1.76 Ab	3.73±0.18 Ac	4.00±0.58 Ac	1.85±0.77 Ab	tr	0.11±0.05 Aa
Biogenic amines							
Tyramine	*L. brevis*	0.22±0.01 Ab	0.18±0.01 Aa	0.19±0.01 Aa	0.18±0.01 Aa	0.16±0.01 Aa	0.16±0.01 Aa
	L. acidophilus	0.22±0.01 Ab	0.19±0.01 Ab	0.21±0.00 Bb	0.20±0.01 Ab	0.14±0.03 Aa	0.17±0.00 Aab
	O. oeni	0.22±0.01 Ac	0.20±0.01 Aabc	0.21±0.00 Bbc	0.18±0.01 Aabc	0.15±0.00 Aab	0.15±0.03 Aa

Spermine	*L. brevis*	0.13±0.04 Aab	0.11±0.01 Ab	0.11±0.01 Aab	0.10±0.01 ABab	0.06±0.02 Aa	0.07±0.00 Bab
	L. acidophilus	0.13±0.04 Aa	0.10±0.01 Aa	0.16±0.01 Bb	0.11±0.01 Ba	0.08±0.02 Aa	0.09±0.00 Ca
	O. oeni	0.13±0.04 Aa	0.20±0.05 Ab	0.08±0.01 Aa	0.08±0.01 Aa	0.07±0.00 Aa	0.06±0.00 Aa
Spermidine	*L. brevis*	0.20±0.02 Ac	0.06±0.00 Aa	0.07±0.03 ABa	0.15±0.02 Bbc	0.09±0.04 Aab	0.04±0.02 ABa
	L. acidophilus	0.20±0.02 Ab	0.19±0.01 Bb	0.05±0.02 Aa	0.08±0.02 Aa	0.08±0.03 Aa	0.08±0.03 Ba
	O. oeni	0.20±0.02 Ad	0.19±0.01 Bcd	0.14±0.02 bc	0.10±0.02 ABab	0.07±0.02 Aa	tr
Cadaverine	*L. brevis*	1.13±0.02 Ab	1.02±0.09 Ab	1.07±0.02 Ab	0.97±0.14 Ab	0.08±0.02 Aa	0.01±0.01 Aa
	L. acidophilus	1.13±0.02 Ac	1.07±0.01 Abc	1.10±0.03 Abc	1.00±0.07 Ab	0.08±0.02 Aa	0.05±0.01 Ba
	O. oeni	1.13±0.02 Ac	1.08±0.01 Ac	1.06±0.01 Abc	1.00±0.04 Ab	0.03±0.00 Aa	0.09±0.01 Ba
Putrescine	*L. brevis*	1.73±0.01 Ab	1.54±0.02 Ab	1.65±0.08 Ab	1.32±0.33 Ab	0.08±0.00 Aa	0.29±0.04 Ba
	L. acidophilus	1.73±0.01 Ac	1.75±0.04 Bc	1.78±0.05 Ac	1.47±0.17 Ab	0.10±0.01 Aa	0.12±0.02 Aa
	O. oeni	1.73±0.01 Ac	1.56±0.07 Abc	1.61±0.04 Abc	1.43±0.19 Ab	0.08±0.00 Aa	0.08±0.00 Aa
Phenethylamine	*L. brevis*	0.99±0.01 Ab	0.89±0.01 Ab	1.00±0.03 ABb	0.93±0.10 Ab	0.11±0.01 Aa	0.10±0.02 Aa
	L. acidophilus	0.99±0.01 Ab	0.99±0.03 Ab	1.03±0.03 Bb	0.95±0.05 Ab	0.10±0.01 Aa	0.08±0.01 Aa
	O. oeni	0.99±0.01 Ac	0.90±0.04 Ab	0.93±0.01 Abc	0.92±0.01 Abc	0.09±0.01 Aa	0.01±0.00 Aa
Histamine	*L. brevis*	3.81±0.21 Aa	3.39±0.55 Aa	4.29±0.17 ABab	4.24±0.66 ABab	4.53±0.41 Aab	5.54±0.01 Bb
	L. acidophilus	3.81±0.21 Aa	5.42±0.42 Bab	6.24±0.31 Bbc	5.23±0.17 Bab	6.91±0.47 Bbc	7.94±1.16 Cc
	O. oeni	3.81±0.21 ABb	3.87±0.67 Ab	3.69±0.61 Ab	3.70±0.17 Ab	4.83±0.18 Ab	0.79±0.17 Aa
Total	*L. brevis*	8.21±0.32 Ab	7.19±0.69 Aab	8.38±0.35 Ab	7.89±1.27 ABab	5.11±0.51 Aa	6.21±0.11 Ba
	L. acidophilus	8.21±0.32 Aab	9.71±0.53 Bb	10.57±0.45 Bb	9.04±0.50 Bab	7.49±0.59 Ba	8.53±1.23 Cab
	O. oeni	8.21±0.32 Ac	8.00±0.86 ABc	7.72±0.70 Ac	7.41±0.45 Ac	5.32±0.21 Ab	1.18±0.21 Aa

Data are mean ± standard deviation of triplicate tests; "tr" represents "trace amount"; Different letters in lower case indicate significant differences with time, whereas different letters in upper case represent significant difference in juice inoculated with different strains at the same sampling interval at $P<0.05$.

Table S4. Content of phenolic acids, flavonols, flavanols, and anthocyanins in bog bilberry juice inoculated with three lactic acid bacteria strains in 14 days.

Compound (mg/L)	Strain	Time (Day)					
		0	1	2	3	7	14
Phenolic acids							
Protocatechuic acid	*L. brevis*	28.37±1.84 Ab	25.38±1.09 Bab	23.54±0.31 Bab	23.21±0.48 Ba	23.22±1.36 Ba	23.95±0.18 Cab
	L. acidophilus	28.37±1.84 Ab	9.47±0.25 Aa	8.73±0.46 Aa	8.15±0.46 Aa	7.02±0.51 Aa	7.58±0.27 Aa
	O. oeni	28.37±1.84 Ab	22.86±0.72 Ba	22.46±0.02 Ba	22.16±0.31 Ba	22.05±0.23 Ba	22.11±0.29 Ba
Chlorogenic acid	*L. brevis*	24.30±0.13 Ac	23.57±1.52 Abc	21.75±0.51 Aabc	20.09±0.29 Aab	18.96±0.76 Aa	19.41±0.25 Aa
	L. acidophilus	24.30±0.13 Ac	23.69±0.35 Ac	23.60±0.44 Ac	21.57±0.05 Bb	20.03±0.18 Aa	20.99±0.16 Bab
	O. oeni	24.30±0.13 Ac	21.88±0.03 Ab	22.08±0.89 Ab	19.74±0.04 Aa	19.09±0.12 Aa	18.75±0.03 Aa
Gallic acid	*L. brevis*	11.71±0.49 Ab	9.75±0.46 Bab	8.76±0.16 Ca	7.80±0.43 Ba	7.83±0.54 Aa	8.19±0.51 Ba
	L. acidophilus	11.71±0.49 Ab	7.55±0.07 Aa	6.74±0.06 Aa	6.32±0.25 Aa	6.25±0.49 Aa	6.56±0.32 Aa
	O. oeni	11.71±0.49 Ab	7.53±0.07 Aa	7.60±0.18 Ba	7.04±0.02 ABa	7.24±0.18 Aa	7.67±0.06 ABa
Caffeic acid	*L. brevis*	3.68±0.36 Ab	2.55±0.33 Bab	2.32±0.20 Ba	2.36±0.23 Ba	2.22±0.29 Ba	1.89±0.11 Ba
	L. acidophilus	3.68±0.36 Ac	1.12±0.09 Ab	0.79±0.01 Aab	0.75±0.12 Aab	0.37±0.03 Aab	0.16±0.01 Aa
	O. oeni	3.68±0.36 Ab	2.87±0.13 Ba	2.69±0.03 Aa	2.63±0.04 Ba	2.39±0.05 Ba	2.13±0.09 Ba
4-Hydroxy-cinnamic acid	*L. brevis*	0.75±0.03 Aa	0.69±0.06 ABa	0.62±0.04 Ba	0.68±0.06 Ba	tr	tr
	L. acidophilus	0.75±0.03 Ac	0.52±0.01 Ab	0.46±0.02 Aab	0.38±0.03 Aa	tr	tr
	O. oeni	0.75±0.03 Ab	0.75±0.03 Bb	0.71±0.02 Bab	0.58±0.05 ABa	tr	tr
3-Hydroxy-cinnamic acid	*L. brevis*	0.37±0.01 Ad	0.21±0.02 Abc	0.27±0.02 Ac	0.19±0.02 Aab	0.18±0.01 Aab	0.13±0.01 Aa
	L. acidophilus	0.37±0.01 Ab	0.17±0.03 Aa	0.23±0.00 Aa	0.25±0.02 Aab	0.22±0.03 Aa	0.24±0.05 Aab
	O. oeni	0.37±0.01 Ab	0.18±0.02 Aa	0.27±0.08 Aab	0.17±0.02 Aa	0.16±0.01 Aa	0.10±0.02 Aa
Ferulic acid	*L. brevis*	0.07±0.00 Ab	0.03±0.01 Aa	0.04±0.01 ABa	tr	tr	tr
	L. acidophilus	0.07±0.00 Ab	0.07±0.01 Bb	0.06±0.01 Bb	0.01±0.01 Aa	tr	tr
	O. oeni	0.07±0.00 Ab	0.03±0.01 Aa	0.03±0.01 Aa	tr	tr	tr

Total	*L. brevis*	69.25±2.08 Ab	62.19±3.49 Bab	57.31±1.24 Ba	54.33±1.33 Ba	52.40±2.83 Ba	53.56±1.02 Ca
	L. acidophilus	69.25±2.08 Ad	42.59±0.10 Ac	40.61±0.92 Abc	37.43±0.65 Aab	33.89±0.88 Aa	35.52±0.29 Aa
	O. oeni	69.25±2.08 Ad	56.10±0.95 Bc	55.84±1.10 Bbc	52.31±0.32 Babc	50.92±0.28 Bab	50.77±0.29 Ba
Flavanols							
Epicatechin	*L. brevis*	18.6±0.94 Ac	18.50±0.99 Ac	15.59±1.20 Ac	9.80±0.02 Ab	8.08±0.25 Aab	6.22±0.06 Aa
	L. acidophilus	18.6±0.94 Ad	18.05±0.02 Acd	15.57±1.07 Ac	11.99±0.13 Bb	8.98±0.47 Aa	8.49±0.09 Ba
	O. oeni	18.6±0.94 Ad	16.30±0.25 Acd	15.58±0.40 Ac	9.41±0.55 Ab	8.27±0.38 Aab	5.76±0.46 Aa
Procyanidin B2							
	L. brevis	5.85±0.15 Ad	4.86±0.35 Ac	4.44±0.20 Ac	2.66±0.02 Ab	1.89±0.21 Aab	1.39±0.13 ABa
	L. acidophilus	5.85±0.15 Ad	5.03±0.05 Acd	4.83±0.42 Ac	3.13±0.10 Ab	2.14±0.09 Aa	1.98±0.02 Ba
	O. oeni	5.85±0.15 Ad	4.89±0.15 Acd	4.53±0.12 Ac	2.92±0.54 Ab	1.94±0.09 Aab	1.31±0.21 Aa
Epigallocatechin	*L. brevis*	1.24±0.08 Ab	1.25±0.06 Ab	1.08±0.04 Ab	0.81±0.01 Aa	0.71±0.04 Aa	0.63±0.01 Aa
	L. acidophilus	1.24±0.08 Ac	1.24±0.02 Ac	1.13±0.05 Abc	1.03±0.07 Aabc	0.87±0.09 Aab	0.76±0.02 Ba
	O. oeni	1.24±0.08 Ad	1.17±0.02 Ad	1.09±0.01 Acd	0.90±0.08 Abc	0.74±0.01 Aab	0.64±0.03 Aa
Gallocatechin	*L. brevis*	1.22±0.08 Ab	1.26±0.00 Ab	1.16±0.05 Bb	0.68±0.03 ABa	0.53±0.04 Aa	0.53±0.01 ABa
	L. acidophilus	1.22±0.08 Ac	1.17±0.09 Ac	0.97±0.06 ABbc	0.79±0.01 Bab	0.76±0.05 Bab	0.57±0.06 Ba
	O. oeni	1.22±0.08 Ad	1.09±0.05 Ad	0.89±0.01 Ac	0.64±0.03 Ab	0.52±0.03 Aab	0.41±0.01 Aa
Catechin	*L. brevis*	0.94±0.04 Ad	0.70±0.01 Bc	0.69±0.06 Ac	0.44±0.04 Ab	0.34±0.01 Aab	0.18±0.02 Aa
	L. acidophilus	0.94±0.04 Ad	0.72±0.01 Bc	0.65±0.09 Abc	0.51±0.03 Ab	0.29±0.03 Aa	0.25±0.01 Aa
	O. oeni	0.94±0.04 Ac	0.61±0.01 Ab	0.60±0.06 Ab	0.50±0.01 Ab	0.28±0.02 Aa	0.22±0.02 Aa
Procyanidin C1	*L. brevis*	0.69±0.10 Aa	0.59±0.31 Aa	0.47±0.20 Aa	tr	tr	tr
	L. acidophilus	0.69±0.10 Aa	0.72±0.02 Aa	0.63±0.25 Aa	tr	tr	tr
	O. oeni	0.69±0.10 Aa	0.57±0.05 Aa	0.56±0.03 Aa	tr	tr	tr
Procyanidin B1	*L. brevis*	0.44±0.01 Ab	0.27±0.01 Aa	0.21±0.05 Aa	tr	tr	tr
	L. acidophilus	0.44±0.01 Ac	0.27±0.03 Ab	0.23±0.02 Aab	0.10±0.06 Aa	tr	tr
	O. oeni	0.44±0.01 Ab	0.24±0.05 Aa	0.20±0.03 Aa	tr	tr	tr
Total	*L. brevis*	28.97±1.03 Ad	27.42±1.74 Acd	23.63±1.70 Ac	14.40±0.07 Ab	11.55±0.32 Aab	8.95±0.19 Aa
	L. acidophilus	28.97±1.03 Ad	27.21±0.02 Acd	24.01±1.91 Ac	17.55±0.27 Bb	13.04±0.60 Aa	12.05±0.15 Ba

	O. oeni	28.97±1.03 Ad	24.87±0.50 Ac	23.43±0.48 Ac	14.38±1.00 Ab	11.74±0.52 Aab	8.34±0.69 Aa

Flavonols							
Myricetin-3-*O*-galactoside	*L. brevis*	93.47±4.03 Ab	78.90±3.87 ABab	79.42±1.76 Aab	76.11±2.74 ABa	72.70±4.15 Aa	74.00±1.01 Aa
	L. acidophilus	93.47±4.03 Ab	84.55±0.44 Bab	82.26±2.00 Aa	80.97±0.92 Ba	79.86±2.71 Aa	73.93±1.54 Aa
	O. oeni	93.47±4.03 Ab	73.09±1.79 Aa	75.31±0.94 Aa	72.48±0.74 Aa	72.58±0.66 Aa	68.94±1.37 Aa
Quercetin-3-*O*-galactoside	*L. brevis*	54.78±0.22 Ac	45.88±2.37 Ab	42.97±0.80 Aab	41.17±1.33 Aab	39.21±2.15 Aab	38.70±0.65 Ba
	L. acidophilus	54.78±0.22 Ad	47.05±0.13 Ac	44.66±1.46 Abc	43.38±0.84 Abc	42.49±1.14 Aab	39.04±0.78 Ba
	O. oeni	54.78±0.22 Ac	40.76±1.24 Ab	40.79±0.23 Ab	39.61±0.42 Ab	39.04±0.32 Ab	35.81±0.41 Aa
Quercetin	*L. brevis*	33.16±1.63 Ab	22.97±2.08 Aa	22.53±1.09 Ba	17.27±1.73 Aa	16.34±1.93 Aa	18.66±0.30 Ba
	L. acidophilus	33.16±1.63 Ad	26.10±0.88 Ac	24.66±1.03 Bbc	18.25±2.54 Aab	18.46±0.19 Aab	12.56±0.30 Aa
	O. oeni	33.16±1.63 Ac	20.62±1.06 Ab	16.08±1.39 Aab	12.88±1.43 Aa	16.43±0.79 Aab	15.89±1.33 ABab
Quercetin-3-*O*-glucuronide	*L. brevis*	26.21±0.57 Aa	24.55±3.07 Aa	24.00±2.08 Aa	25.69±1.50 Ba	24.34±1.11 Ba	22.15±1.98 Aa
	L. acidophilus	26.21±0.57 Ac	21.81±0.16 Ab	21.44±0.77 Ab	20.59±0.58 Aab	20.32±0.70 Aab	18.71±0.23 Aa
	O. oeni	26.21±0.57 Ab	18.25±0.66 Aa	18.75±0.42 Aa	17.23±0.56 Aa	18.62±0.45 Aa	17.33±0.24 Aa
Syringetin-3-*O*-galactoside	*L. brevis*	13.69±0.27 Ab	11.54±0.63 Aa	10.71±0.21 Aa	10.74±0.39 Aa	10.26±0.63 Aa	10.04±0.13 ABa
	L. acidophilus	13.69±0.27 Ac	11.71±0.14 Ab	11.22±0.39 Aab	11.12±0.14 Aab	10.7±0.23 Aab	10.49±0.21 Ba
	O. oeni	13.69±0.27 Ac	10.63±0.31 Ab	10.53±0.10 Aab	10.06±0.10 Aab	9.67±0.14 Aa	9.73±0.13 Aab
Myricetin	*L. brevis*	7.89±0.68 Ab	6.21±0.62 Aab	6.10±0.28 ABab	5.11±0.44 Aa	5.44±0.01 Ba	5.35±0.12 Aa
	L. acidophilus	7.89±0.68 Ac	6.84±0.11 Abc	6.81±0.31 Bbc	5.56±0.44 Aab	5.50±0.12 Bab	4.33±0.42 Aa
	O. oeni	7.89±0.68 Ab	5.11±0.51 Aa	4.98±0.18 Aa	4.30±0.29 Aa	5.03±0.03 Aa	4.62±0.25 Aa
Myricetin-3-*O*-glucoside	*L. brevis*	3.91±0.01 Aab	4.27±0.25 ABb	3.81±0.02 Bab	3.43±0.21 Aab	3.18±0.29 Aa	3.10±0.12 Aa

	L. acidophilus	3.91±0.01 Ac	4.50±0.14 Bd	3.74±0.14 Bc	3.46±0.09 Abc	3.15±0.20 Aab	2.72±0.04 Aa
	O. oeni	3.91±0.01 Ac	3.48±0.12 Abc	3.18±0.13 Aab	2.85±0.17 Aa	3.33±0.16 Aabc	2.82±0.11 Aa
Isorhamnetin-3-*O*-glucoside	*L. brevis*	3.74±0.13 Ad	3.31±0.14 ABcd	3.14±0.03 Abc	2.94±0.10 Aabc	2.70±0.20 Aab	2.57±0.05 Aa
	L. acidophilus	3.74±0.13 Ad	3.52±0.03 Bcd	3.22±0.12 Abc	3.04±0.08 Ab	2.86±0.05 Aab	2.61±0.09 Aa
	O. oeni	3.74±0.13 Ad	2.97±0.12 Ac	2.98±0.01 Ac	2.75±0.02 Abc	2.54±0.06 Aab	2.38±0.04 Aa
Syringetin-3-*O*-glucoside	*L. brevis*	3.52±0.03 Ab	3.22±0.20 Aab	3.21±0.02 Bab	2.90±0.16 Aab	2.61±0.21 Aa	2.59±0.06 Aa
	L. acidophilus	3.52±0.03 Ab	3.33±0.03 Ab	2.87±0.08 Aa	2.97±0.09 Aa	2.92±0.04 Aa	2.82±0.04 Ba
	O. oeni	3.52±0.03 Ac	2.95±0.04 Ab	2.74±0.07 Aab	2.65±0.09 Aa	2.61±0.08 Aa	2.57±0.02 Aa
Laricitrin	*L. brevis*	1.60±0.20 Ab	1.18±0.17 Aab	1.20±0.05 Bab	0.88±0.11 Aa	0.88±0.03 Aa	0.97±0.05 Aa
	L. acidophilus	1.60±0.20 Ac	1.39±0.03 Abc	1.31±0.11 Bbc	1.01±0.14 Aab	0.93±0.03 Aab	0.70±0.10 Aa
	O. oeni	1.60±0.20 Ab	1.10±0.05 Aa	0.86±0.06 Aa	0.68±0.03 Aa	0.84±0.04 Aa	0.75±0.08 Aa
Dihydroquercetin	*L. brevis*	1.54±0.02 Ad	0.83±0.04 Ac	0.76±0.03 Ac	0.41±0.01 Ab	0.39±0.04 Bab	0.24±0.03 Ba
	L. acidophilus	1.54±0.02 Ad	0.80±0.02 Ac	0.73±0.09 Ac	0.41±0.10 Ab	0.12±0.02 Aa	0.10±0.02 Aa
	O. oeni	1.54±0.02 Ac	0.69±0.08 Ab	0.66±0.03 Ab	0.37±0.05 Aa	0.34±0.02 Ba	0.19±0.00 ABa
Isorhamnetin	*L. brevis*	1.42±0.16 Ab	1.19±0.06 Aab	1.20±0.01 Bab	0.96±0.08 Aa	0.90±0.07 Aa	0.97±0.01 Aa
	L. acidophilus	1.42±0.16 Ac	1.31±0.04 Abc	1.17±0.02 Babc	1.01±0.09 Aab	1.03±0.01 Aab	0.85±0.03 Aa
	O. oeni	1.42±0.16 Ab	1.01±0.13 Aab	0.91±0.05 Aa	0.70±0.03 Aa	0.95±0.02 Aa	0.90±0.05 Aa
Total	*L. brevis*	244.93±5.76 Ab	204.05±13.49 Aa	199.05±6.32 ABa	187.61±7.68 Aa	178.94±9.86 Aa	179.33±4.12 Ba
	L. acidophilus	244.93±5.76 Ad	212.92±1.44 Ac	204.07±6.38 Bbc	191.77±5.83 Aabc	188.35±5.21 Aab	168.87±2.34 ABa
	O. oeni	244.93±5.76 Ac	180.68±5.29 Ab	177.76±2.71 Aab	166.58±2.77 Aab	172.00±1.46 Aab	161.93±3.04 Aa

Data are mean ± standard deviation of triplicate tests; Different letters in lower case indicate significant differences with time, whereas different letters in upper case represent significant difference in juice inoculated with different strains at the same sampling interval at $P<0.05$
